# 1,25-Dihydroxyvitamin D_3_ attenuates endotoxin-induced production of inflammatory mediators by inhibiting MAPK activation in primary cortical neuron-glia cultures

**DOI:** 10.1186/s12974-015-0370-0

**Published:** 2015-08-12

**Authors:** Ya-Ni Huang, Yi-Jung Ho, Chien-Cheng Lai, Chien-Tsai Chiu, Jia-Yi Wang

**Affiliations:** Department of Nursing, Hsin Sheng Junior College of Medical Care and Management, Taoyuan City, Taiwan; Institute of Preventive Medicine, National Defense Medical Center, Taipei, Taiwan; Graduate Institute of Life Sciences, National Defense Medical Center, Taipei, Taiwan; Division of Orthopedics, Department of Surgery, Far Eastern Memorial Hospital, New Taipei City, Taiwan; Department of Neurosurgery, En Chu Kong Hospital, New Taipei City, Taiwan; Graduate Institute of Medical Sciences and Department of Physiology, College of Medicine, Taipei Medical University, 250 Wu-Hsing Street, Taipei, 110 Taiwan

## Abstract

**Background:**

Neuroinflammation occurs in insulted regions of the brain and may be due to reactive oxygen species (ROS), nitric oxide (NO), cytokines, and chemokines produced by activated glia. Excessive production of neurotoxic molecules causes further neuronal damage. Low levels of vitamin D_3_ are a risk factor for various brain diseases.

**Methods:**

Using the bacterial endotoxin, lipopolysaccharide (LPS), to induce neuroinflammation in primary cortical neuron-glia cultures, we investigated how 1,25-dihydroxyvitamin D3 (1,25(OH)_2_D_3_) affected neuroinflammation.

**Results:**

LPS (100 ng/ml) induced the accumulation of nitrite and the production of ROS, interleukin (IL)-6, and macrophage inflammatory protein (MIP)-2 in time-dependent manners. Inhibition of p38 and extracellular signal-regulated kinase (ERK) but not c-Jun N-terminal kinase (JNK) mitogen-activated protein kinase (MAPK) by 20 μM of SB203580, PD98059, and SP600125, significantly reduced LPS-induced ROS production, NO accumulation, and inducible NO synthase (iNOS) expression, respectively. LPS-induced IL-6 and MIP-2 were significantly attenuated by inhibition of p38, ERK, and JNK MAPK. Cotreatment with 1,25(OH)_2_D_3_ attenuated LPS-induced ROS production, NO accumulation, and iNOS expression in concentration-dependent manners. 1,25(OH)_2_D_3_ also reduced LPS-induced production of IL-6 and MIP-2. Similarly, iNOS, IL-6, and MIP-2 mRNA expression in cells treated with LPS significantly increased, whereas this effect was attenuated by 1,25(OH)_2_D_3_. Moreover, LPS-induced phosphorylation of p38, ERK, and JNK MAPK was significantly inhibited by 1,25(OH)_2_D_3_.

**Conclusions:**

Our findings indicate that 1,25(OH)_2_D_3_ reduced the LPS-stimulated production of inflammatory molecules in neuron-glia cultures by inhibiting MAPK pathways and the production of downstream inflammatory molecules. We suggest that 1,25(OH)_2_D_3_ can be used to alleviate neuroinflammation in various brain injuries.

## Introduction

1,25-Dihydroxyvitamin D_3_ (1,25-(OH)_2_D_3_) is a secosteroid hormone, synthesized through a multistep process, which begins in the skin and is completed in the kidneys. Ultraviolet light photocatalyzes conversion of the precursor, 7-dehydrocholesterol, to vitamin D_3_ or cholecalciferol, which has no biological activity until its conversion to the active form, 1,25-(OH_2_)D_3_ [1]. The activated vitamin D metabolite has many roles in regulating homeostasis (e.g., calcium homeostasis and maintenance) throughout the body. 1,25-(OH)_2_D_3_ has effects on the classic target organs (e.g., bones, intestines, and kidneys) and stimulates calcium transport from these organs to the blood. A growing body of evidence has demonstrated that 1,25-(OH)_2_D_3_ plays an important role in non-classical actions such as regulating immune function [2]. It is known that 1,25-(OH)_2_D_3_, as a potent neuromodulator of the immune system, exerts marked effects on neural cells [3]. 1,25-(OH)_2_D_3_ was shown to regulate neurotrophic factors in the brain, including nerve growth factors (NGFs) [4], neurotrophin 3 (NT3) [5], and glial cell line-derived neurotrophic factor (GDNF) [6]. Additionally, 1,25-(OH)_2_D_3_ increases expressions of microtubule-associated protein-2, growth-associated protein-43 [7], and neurite outgrowth [8] in cultured neurons, indicating that 1,25-(OH)_2_D_3_ may also affect neuronal plasticity processes.

Clinical studies suggested that a vitamin D insufficiency is associated with an increased risk of brain insults such as Alzheimer’s disease (AD) [9], Parkinson’s disease [10], and ischemic brain injury [6]. In animal studies, a vitamin D deficiency exacerbated stroke brain injury and dysregulated ischemia-induced inflammation [11], whereas administration of 1,25-(OH)_2_D_3_ reduced ischemia-induced brain damage through upregulating GDNF expression [6]. Pretreatment with 1,25-(OH)_2_D_3_ attenuated hypokinesia and dopaminergic neurotoxicity induced by 6-OHDA in rats [12]. Moreover, 1,25-(OH)_2_D_3_ increased secretion of anti-inflammatory cytokines and reduced secretion of proinflammatory cytokines [4, 5, 13], suggesting that 1,25-(OH)_2_D_3_ may be neuroprotective and may regulate neuroinflammation in the brain. However, the underlying mechanisms of vitamin D’s effect on neuroinflammation remain unclear.

Neuroinflammation is a common mechanism and plays a crucial role in the pathogenesis of various nerve diseases. Initiation of a neuroinflammatory response involves a complex interplay of glia. Activated glial cells, mainly astrocytes and microglia, are thus histopathological hallmarks of neurologic diseases. Inflammatory mediators (e.g., nitric oxide (NO), reactive oxygen species (ROS), proinflammatory cytokines, and chemokines) released by activated glia are neurotoxic and can cause neuronal damage [14]. It is known that lipopolysaccharide (LPS), a gram-negative bacterial cell wall endotoxin, can activate glia through Toll-like receptors, triggering downstream signaling, such as mitogen-activated protein kinases (MAPKs). Three major MAPK subfamilies have been described: p38, extracellular signal-regulated kinase (ERK), and c-Jun N-terminal kinase (JNK). Activation of MAPK pathways by LPS initiates neuroinflammatory cascades characterized by activation of glia and increasing production of inflammatory mediators including ROS, NO, cytokines, and chemokines [15–17]. Therefore, controlling activated glia can be a therapeutic strategy for neuroinflammation.

Studying the protective roles of antioxidant compounds in inhibiting the inflammatory response in brain diseases is an important vista for further research and clinical applications. Using cortical neuron-glia cultures, we investigated how 1,25-(OH)_2_D_3_ affected LPS-induced neuroinflammatory responses, by exploring whether the effects of 1,25-(OH)_2_D_3_ are mediated through MAPK pathways.

## Materials and methods

### Chemical reagents and antibodies

1,25-(OH)_2_D_3_ (SI-D1530) and LPS (L3129) were purchased from Sigma-Aldrich (St. Louis, MO). The p38 MAPK inhibitor, SB203580, ERK inhibitor, PD98059, JNK inhibitor, SP600125, iNOS, and β-actin were purchased from Calbiochem (San Diego, CA). Antibodies against ERK, p38, JNK, phosphorylated (p)-p38, p-ERK (p-p42/p44), and p-JNK (p-p46/p54) were purchased from Cell Signaling Technology (Beverly, MA). Antibodies against microtubule-associated protein-2 (MAP-2) and glial fibrillary acid protein (GFAP) were purchased from Chemicon (Temecula, CA). Antibody against ED1 was purchased from Serotec (Bicester, UK). Antibodies against oligodendrocyte marker 4 (O4), fibronectin 1 (FN1), and rat endothelial cell antigen (RECA-1) were purchased from R&D systems (Minneapolis, MN), Bioworld Technology (MN, USA), and Abcam (Cambridge, MA), respectively.

### Primary rat cortical neuron-glia cultures

Primary neuron-glia cultures were prepared from the cerebral cortex of 1-day-old neonatal Sprague–Dawley rats, as previously described [18–31]. All animal procedures were approved by the Institutional Animal Care and Use Committee of Taipei Medical University (Taipei, Taiwan) (permit no.: LAC-101-0249). These procedures were performed according to the National Institutes of Health Guidelines for the Care and Use of Laboratory Animals. After the rats were sacrificed, their brains were quickly removed aseptically, and the blood vessels and meninges were discarded. Cerebral cortices were dissected under sterile conditions and kept on ice in Hank’s solution (without Ca^2+^ or Mg^2+^). Subsequently, cortical cells were dissociated by trituration using a pipette. Cells were centrifuged (1500 rpm for 5 min) and resuspended in 10 % fetal bovine serum/Dulbecco’s modified Eagle’s medium (Gibco BRL, Grand Island, NY). To each well of 24-well culture plates was seeded 5 × 10^5^ cells in 0.5 ml of culture medium. Cells were incubated at 37 °C and 5 % CO_2_ at a humidity of 95 % and used for experiments starting on day 14 of cultivation in vitro. The percentage cell composition was determined by immunostaining, followed by cell counting. The neuron-glia cultures consisted of approximately 35 % neurons, 54 % astrocytes, and 6 % microglia. In addition, cultures also consisted of approximately 4 % of fibroblasts and a small percentage (<1 %) of other cells including oligodendrocytes, and endothelial cells (pictures not shown).

### Immunocytochemistry

Cultures were fixed in 4 % paraformaldehyde, as previously described [18]. Background staining was reduced by blocking nonspecific binding sites with 10 % goat serum for 1 h at room temperature. After washing with phosphate-buffered saline (PBS), endogenous peroxidase activity was quenched by incubation with a 3 % H_2_O_2_ solution in PBS. Cultures were washed again and then incubated overnight with the appropriate primary antibodies (rabbit anti-MAP-2, 1:500, Chemicon; rabbit anti-GFAP, 1:1000, Chemicon; mouse anti-ED1, 1:500, Serotec; mouse anti-O4, 1:300, R&D system; rabbit-anti fibronectin 1, 1:500, Bioworld Technology; mouse-anti RECA-1, 1:300, Abcam) at 4 °C. Cells were washed and visualized using the avidin-biotin peroxidase complex method (ABC Elite kit; Vector Laboratories, Burlingame, CA). Images were viewed on an inverted Olympus IX 70 microscope (Tokyo City, Japan) equipped with a cooled CCD camera and SPOT advanced software (Diagnostic Instruments, Sterling Heights, MI).

### Cell-type identification and counting

Types of cells present in the culture were identified by immunocytochemical staining using cell-specific markers (MAP-2 for neurons, GFAP for astrocytes, ED1 for microglia, FN1 for fibroblasts, O4 for oligodendrocytes, and RACE-1 for endothelial cells). Images of immunostained-positive cells in each well (in five randomly selected fields with a calibrated area) were captured as digital micrographs, and cells were counted by an observer who was blinded to our study and confirmed by the experiment operator.

### Measurement of ROS

ROS production was detected using the fluorochrome, 2′,7′-dichlorofluorescin diacetate (DCF-DA). Treated cells were incubated with 30 μM DCF-DA in cultured medium for 20 min at 37 °C. After incubation, the culture medium was removed, and then 500 μl of culture medium was added to each culture well. Plates were read with a fluorometric microplate reader at 485/500 nm [23]. Production of ROS was calculated as the maximum DCF fluorescence following incubation (20 min). The value of DCF fluorescence is expressed in arbitrary fluorescence units (AU).

### Determination of NO accumulation

Nitrite accumulation was measured using the Griess reaction in culture media. After treatment, culture medium was collected for measurement of nitrite. Briefly, 50 μl of culture medium was mixed with an equal volume of Griess reagent. The absorbance was detected at 540 nm by a microplate reader (Molecular Devices, Menlo Park, CA).

### Determination of IL-6 and macrophage inflammatory protein (MIP)-2 release

Levels of IL-6 and MIP-2 secreted into the culture media were measured by enzyme-linked immunosorbent assay (ELISA) kits (BioSource International) according to the manufacturer’s protocol.

### Western blotting

Whole cell lysates were prepared from cultured neuronal/glial cells after 24 h of desired treatments. Treated cells were harvested, washed with PBS, and lysed in protein extraction buffer (Mammalian Cell-PE LBTM, Geno Technology) containing protease and phosphatase inhibitors (Complete Mini, Roche Diagnostics, Indianapolis, IN). Cell debris was removed by centrifugation at 12,000 rpm for 15 min at 4 °C, and the supernatant was collected for storage at −80 °C or to perform a Western blot analysis. Equal amounts of protein were separated on 10~15 % sodium dodecylsulfate polyacrylamide gel electrophoresis (SDS-PAGE). Proteins were electroblotted onto polyvinylidene difluoride membranes for 120 min under 100 V (PerkinElmer Life Sciences). Membranes were blocked with 5 % non-fat milk for 1 h, and then incubated overnight at 4 °C with the indicated antibodies, including those for inducible NO synthase (iNOS), p38, phosphorylated (p)-p38, p42/44, p-p42/44, p46/54, and p-p46/54 (1:1000 dilution) followed by an appropriate secondary antibody (at a 1:2 × 10^4^ dilution) for 1 h at room temperature. Signals were visualized using enhanced chemiluminescent detection reagents (PerkinElmer Life Sciences). The membrane was then stripped and reprobed with an antibody specific for β-actin (at a 1:10^4^ dilution) to ensure the accuracy of each loading. The protein signal intensity was quantified by a BioImaging System (Level Biotechnology) and normalized with the corresponding β-actin intensity.

### Real-time reverse-transcription polymerase chain reaction (RT-PCR) assay

Total RNA was extracted from treated cells using the TRIzol® reagent (Invitrogen Life Technologies, Paisley, Scotland) according to the manufacturer’s protocol. Three micrograms of total RNA was reverse-transcribed into complementary (c)DNA using the Rever Tra Ace-α First-strand cDNA Synthesis Kit (TOYOBO Life Science, Japan). The resulting cDNA was incubated with the Rotor-Gene SYBR Green kit (QIAGEN Biosystems, CA) and primers for iNOS (sense, 5′-TTCTTTGCTTCTGTGCTAATGC-3′ and antisense, 5′-ATACTGTTCCATGCAGACAACC-3′); IL-6 (sense, 5′-TTCTTGGGACTGATGTTGTTGAC-3′ and antisense, 5′-AATTAAGCCTCCGACTTGTGAAG-3′); MIP-2 (sense, 5′-AAACTGCACCCAGGAAGCC-3′ and antisense, 5′-ACAGTGAGCTGGCCAATGC-3′); and β-actin (sense, 5′-GACCCAGATCATGTTTGAGACCTTC-3′ and antisense, 5′-GGTGACCGTAACACTACCTGAG-3′). For the semiquantitative analysis, we performed 40 amplification cycles (of denaturation at 95 °C for 5 s and annealing at 60 °C for 10 s) on a Rotor-Gene Q PCR Detection System (QIAGEN Biosystems). A melting curve analysis and sequencing data were used to confirm the specificity of the PCR products. Levels of iNOS, IL-6, and MIP-2 messenger (m)RNAs were normalized to those of β-actin and were then expressed as values relative to the control using the comparative threshold cycle (Ct) method.

### 3-(4,5-Dimethylthianol-2-yl)-2,5 diphenyl tetrazolium bromide (MTT) reduction assay

To examine cell viability, a colorimetric MTT reduction assay was performed as previously described [24]. After a 2-h treatment, the MTT reagent (0.5 mg/ml MTT in PBS) was added to each culture well, and cultured cells were incubated at 37 °C for 40 min. The culture medium was then aspirated from each well, and cells were dissolved by adding DMSO. The absorbance was measured at a test wavelength of 570 nm and a reference wavelength of 630 nm using a microplate reader (Molecular Devices).

### Measurement of lactate dehydrogenase (LDH) release

Cytotoxicity was detected in culture media using an assay of LDH release, as an index of cell injury [18, 25]. LDH activity (units/min) was calculated from the slope of the decrease in the optical density at 340 nm over a 3-min period. The level of LDH was expressed as a percentage of values relative to PBS-treated control (Cont.) cultures.

### Data analysis

All values are presented as the mean ± standard error of the mean (SEM). Experimental data were assessed using a one-way analysis of variance (ANOVA) followed by the Newman-Keuls’ test using the SigmaStat program (Jandel Scientific, San Rafael, CA). Significance was set at *p* < 0.05.

## Results

### LPS-induced ROS production, nitrite accumulation, and the release of proinflammatory mediators

**Fig. 1:**
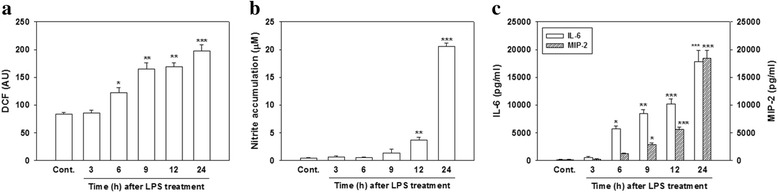
Effect of lipopolysaccharide (LPS) on reactive oxygen species (ROS) production, nitrite accumulation, and interleukin (IL)-6 and macrophage inflammatory protein (MIP)-2 production. Cultured cells were treated with LPS (100 ng/ml) for the indicated time periods. **a** ROS production by cells was detected by DCF fluorescence. The y-axis of the graph is expressed in DCF fluorescent arbitrary units (AU). **b** After treatment, the culture medium was collected to analyze levels of nitrite accumulation. **c** The culture medium was collected to analyze levels of IL-6 and MIP-2. Data are expressed as the mean ± SEM (*n* = 5 in each group). *p < 0.05, **p < 0.01, ***p < 0.001 vs. the control (Cont.)

To examine whether LPS elicited neuroinflammatory responses in cultured neurons/glia, we first examined the time course of LPS-induced ROS production, nitrite accumulation, and the release of IL-6 (a cytokine) and MIP-2 (a chemokine). As shown in Fig. 1a, treating cells with LPS (100 ng/ml) for the indicated periods (3, 6, 9, 12, and 24 h) induced ROS production in a time-dependent manner. ROS production significantly increased in cultured cells exposed to LPS for 6 h (*p* < 0.05) and continued to increase from 12 to 24 h (*p* < 0.01 and *p* < 0.001, respectively). NO production, as reflected by nitrite accumulation, significantly increased in cultured cells exposed to LPS for 12 h (*p* < 0.01) and continued to increase from 12 to 24 h (*p* < 0.001; Fig. 1b). Additionally, IL-6 significantly increased in cultured cells exposed to LPS for 6 h (*p* < 0.05) and remained elevated for up to 24 h (*p* < 0.001; Fig. 1c). Similarly, we also found that MIP-2 increased in cultured cells exposed to LPS for 9 h (*p* < 0.05) and remained elevated for up to 24 h (*p* < 0.001; Fig. 1c). As expected, LPS successfully initiated a neuroinflammatory response in our neuronal/glial coculture system.

### Involvement of MAPK signaling pathways in LPS-induced production of proinflammatory mediators

**Fig. 2:**
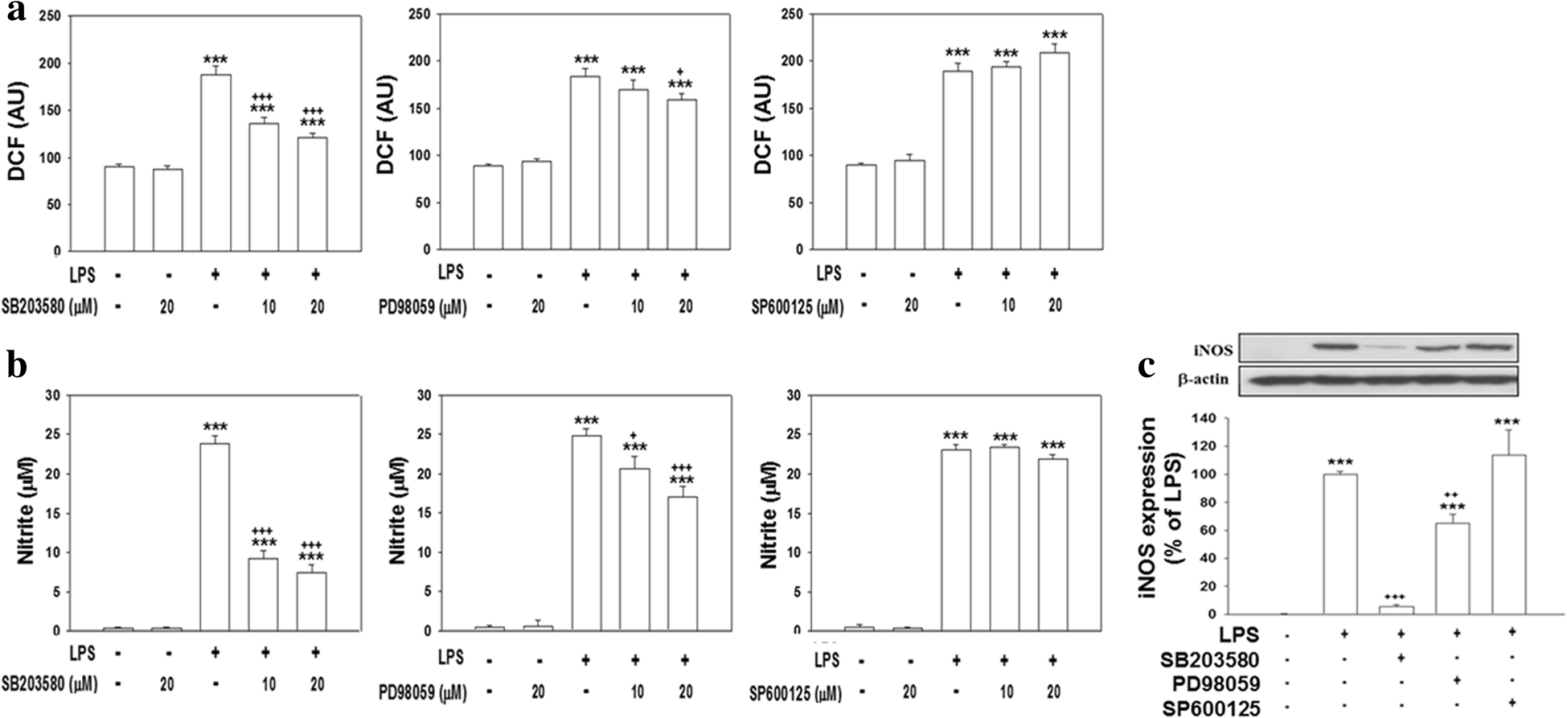
Effect of mitogen-activated protein kinase (MAPK) inhibitors on lipopolysaccharide (LPS)-induced reactive oxygen species (ROS) production, nitrite accumulation, and inducible nitric oxide synthase (iNOS) expression. Cultured cells were treated with various concentrations (10 and 20 μM) of SB203580 (a p38 inhibitor), PD98059 (an extracellular signal-regulated kinase (ERK) inhibitor), or SP600125 (a c-Jun N-terminal kinase (JNK) inhibitor), and then exposed to LPS (100 ng/ml) for 24 h. **a** ROS production by cells was detected by DCF fluorescence. The y-axis of the graph is expressed in DCF fluorescent arbitrary units (AU). **b** The culture medium was collected to analyze levels of nitrite accumulation. **c** After treatment, cells were harvested for a Western blot analysis. Levels of iNOS were normalized to β-actin, and then quantified. The iNOS expression induced by LPS was set to 100 % expression. Data are expressed as the mean ± SEM (*n* = 4 or 5 in each group). ***p < 0.001 vs. the control (Cont.); ^+^ *p* < 0.05, ^++^ *p* < 0.01, ^+++^ *p* < 0.001 vs. LPS

To examine whether MAPKs (e.g., p38, ERK, and JNK) are involved in the production of LPS-initiated proinflammatory mediators, we treated cells with various concentrations (10 and 20 μM) of MAPK inhibitors and observed how these inhibitors affected levels of ROS production, nitrite accumulation, iNOS expression, and IL-6 and MIP2 production. In the absence of LPS, neither SB203580 (a p38 inhibitor), PD98059 (an ERK inhibitor), nor SP600125 (a JNK inhibitor) at 20 μM significantly affected ROS production (Fig. 2a). Cotreatment of 10 or 20 μM SB203580 with LPS for 24 h significantly attenuated LPS-induced ROS production; and 20 μM SB203580 more completely inhibited ROS production compared to 10 μM; however, the difference was non-significant. Cotreatment of 20 μM but not 10 μM PD98059 with LPS produced a significant reduction in ROS (*p* < 0.05). However, cotreatment of 10 or 20 μM JNK with LPS did not reduce ROS production (Fig. 2a). Figure 2b shows that cells treated with 10 or 20 μM SB203580 and LPS exhibited significantly attenuated nitrite accumulation, and 20 μM more completely inhibited this accumulation compared to 10 μM. Both SB203580 and PD98059 but not SP600125 at 20 μM significantly reduced LPS-induced nitrite accumulation (*p* < 0.001). Regarding iNOS induction, treatment with 20 μM of each inhibitor (SB203580, PD98059, and SP600125) alone had no effect (data not shown). Western blot analyses indicated that cotreatment with SB203580 or PD98059 and LPS for 24 h significantly attenuated the level of LPS-induced iNOS expression (*p* < 0.001 and *p* < 0.01, respectively), whereas cells cotreated with the JNK inhibitor and LPS exhibited no effect (Fig. 2c).

**Fig. 3:**
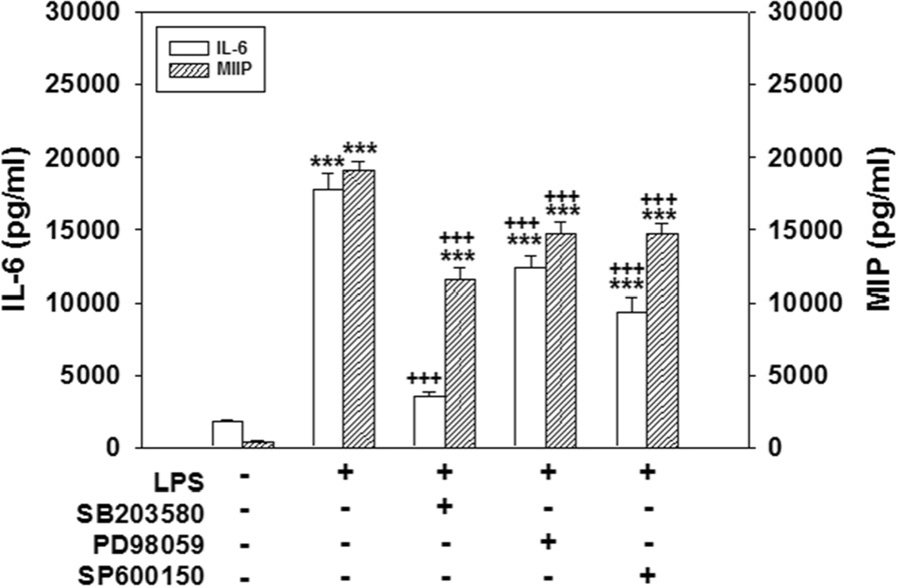
Effect of mitogen-activated protein kinase (MAPK) inhibitors on lipopolysaccharide (LPS)-induced interleukin (IL)-6 and macrophage inflammatory protein (MIP)-2 production. Cultured cells were treated for 24 h with 20 μM SB203580, 20 μM PD98059, or 20 μM SP600125 and were then exposed to LPS (100 ng/ml). The culture medium was collected to analyze levels of IL-6 and MIP-2. Data are expressed as the mean ± SEM *(n* = 4 in each group). ***p < 0.001 vs. the control (Cont.); ^+++^ *p* < 0.001 vs. LPS

Additionally, cotreating cells with SB203580, PD98059, or SP600125 and LPS for 24 h significantly attenuated the release of IL-6 (*p* < 0.001) and MIP-2 (*p* < 0.001) compared to the release in cultures treated with LPS alone (Fig. 3). Levels of IL-6 and MIP-2 production in cultures treated with SB203580, PD98059, or SP600125 alone did not significantly differ from those produced in control cultures (data not shown). These results suggest that activation of p38, ERK, and JNK MAPKs is involved in LPS-induced ROS production, nitrite accumulation, and proinflammatory mediator release.

### 1,25-(OH)_2_D_3_ suppressed LPS-induced ROS production, nitrite accumulation, iNOS expression, and the release of inflammatory mediators without causing cell death

**Fig. 4:**
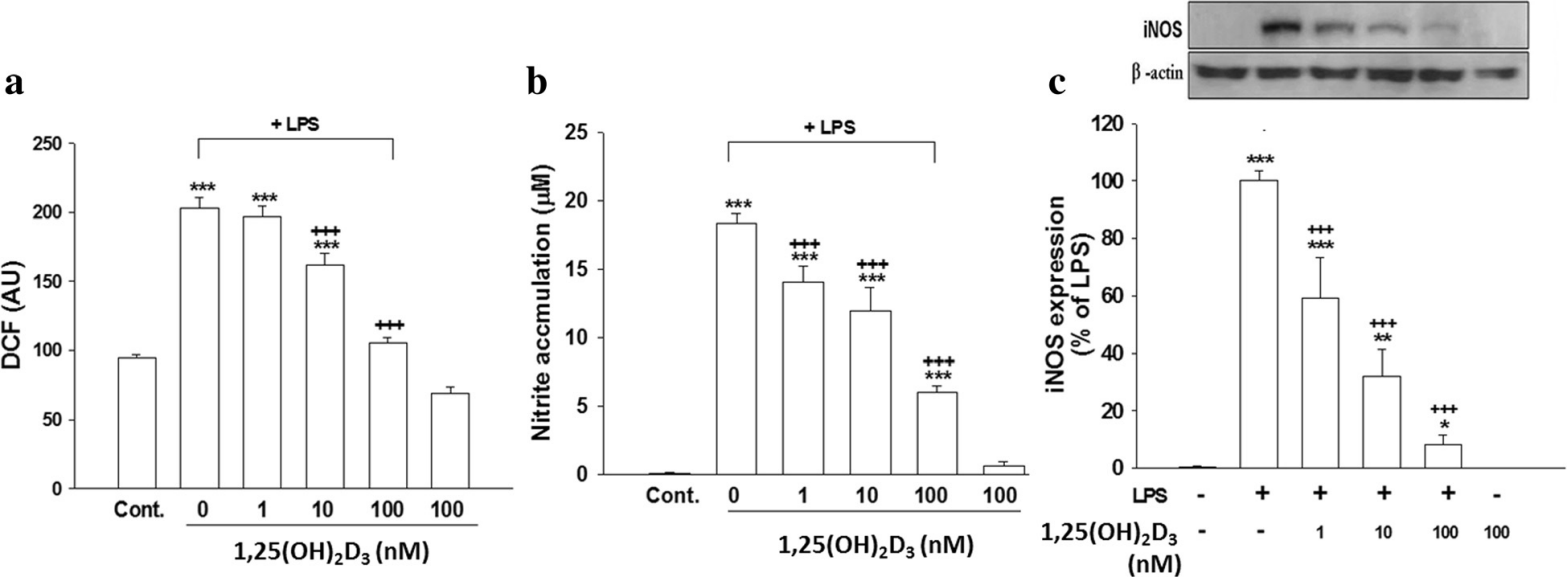
Effects of 1,25(OH)_2_D_3_ on levels of lipopolysaccharide (LPS)-induced reactive oxygen species (ROS) production, nitrite accumulation, and inducible nitric oxide synthase (iNOS) expression. Cultured cells were untreated (PBS) or treated with 1,25(OH)_2_D_3_ (100 nM), LPS (100 ng/ml), or various concentration of 1,25(OH)_2_D_3_ (1, 10, or 100 nM) plus LPS for 24 h. **a** ROS production by cells was detected by DCF fluorescence. The y-axis of the graph is expressed in DCF fluorescent arbitrary units (AU). **b** The culture medium was collected to analyze levels of nitrite accumulation. **c** After treatment, cells were harvested to analyze levels of iNOS protein expression using Western blotting. Levels of iNOS were normalized to β-actin and then quantified. iNOS expression induced by LPS was set to 100 % expression. Data are expressed as the mean ± SEM (*n* = 5 in each group). *p < 0.05, **p < 0.01, ***p < 0.001 vs. the control (Cont.); ^+++^ *p* < 0.001 vs. LPS

**Fig. 5:**
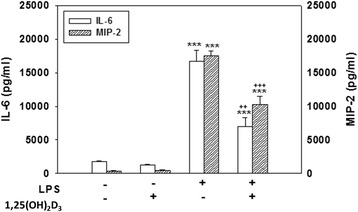
Effect of 1,25(OH)_2_D_3_ on lipopolysaccharide (LPS)-induced interleukin (IL)-6 and macrophage inflammatory protein (MIP)-2 release. Cultured cells were untreated (PBS) or treated with 1,25(OH)_2_D_3_ (100 nM), LPS (100 ng/ml), or 1,25(OH)_2_D_3_ plus LPS for 24 h, and then the culture medium was collected to analyze levels of IL-6 and MIP-2. Data are expressed as the mean ± SEM (*n* = 4 in each group). ***p < 0.001 vs. the control (Cont.); ^++^ *p* < 0.01, ^+++^ *p* < 0.001 vs. LPS

**Fig. 6:**
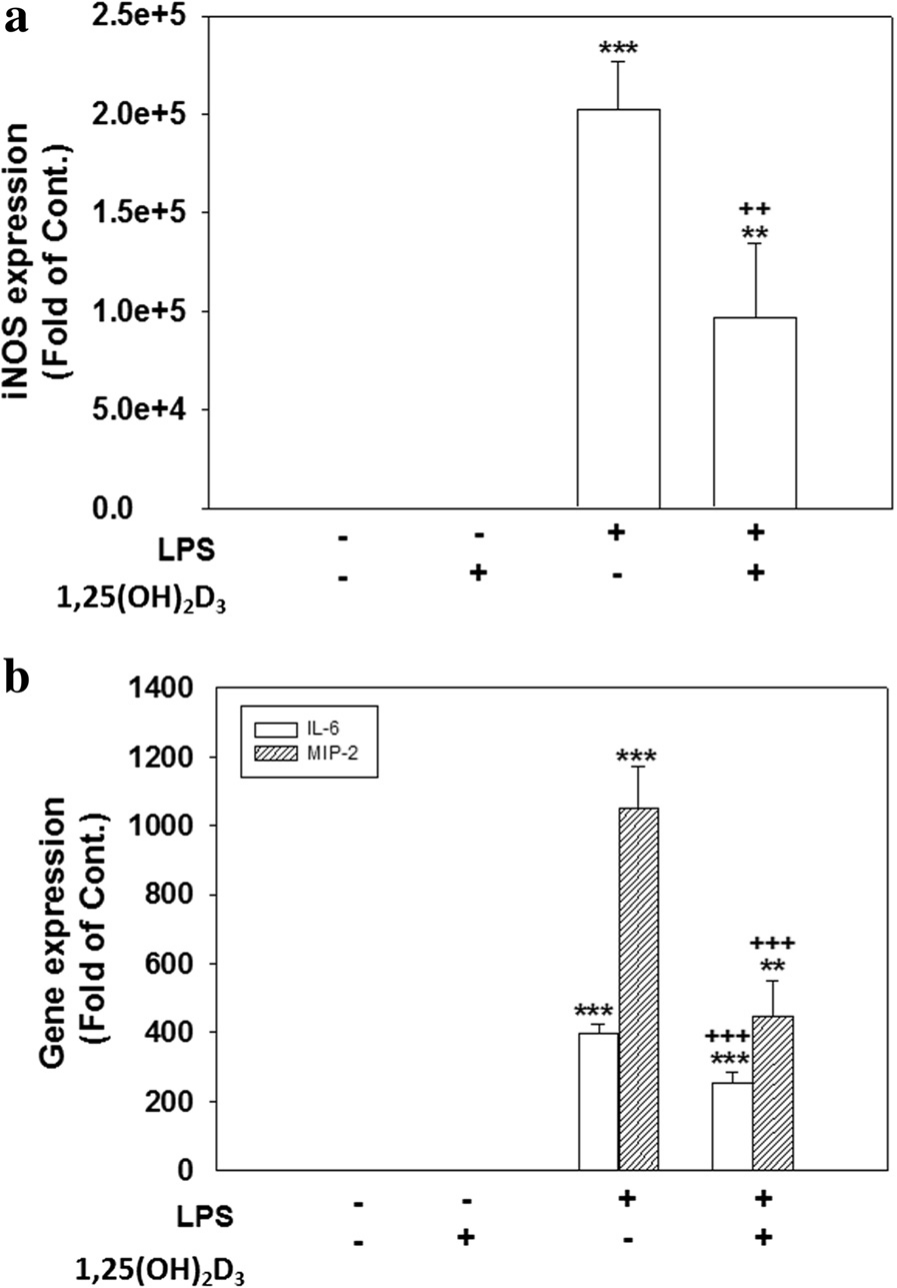
Effect of 1,25(OH)_2_D_3_ on lipopolysaccharide (LPS)-induced inducible nitric oxide synthase (iNOS), interleukin (IL)-6, and macrophage inflammatory protein (MIP)-2 gene expressions. Cultured cells were untreated (PBS) or treated with 1,25(OH)_2_D_3_ (100 nM), LPS (100 ng/ml), or 1,25(OH)_2_D_3_ plus LPS for 24 h. After treatment, cultured cells were harvested to conduct an RT-PCR assay of iNOS (**a**), IL-6, and MIP-2 (**b**) mRNA levels. Expression levels of iNOS, IL-6, and MIP-2 mRNAs were normalized to β-actin. Data are expressed as the mean ± SEM (*n* = 4 in each group). **p < 0.01 and ***p < 0.001 vs. the control (Cont.); ^++^ *p* < 0.01, ^+++^ *p* < 0.001 vs. LPS

1,25-(OH)_2_D_3_, a potent functional hormone, is one of the important nuclear steroid transcription regulators that regulate the transcription of a large number of genes. We examined whether 1,25-(OH)_2_D_3_ could attenuate an experimental neuroinflammatory response produced using LPS in a neuron-glia culture model. Exposing cells to LPS for 24 h significantly enhanced ROS production (*p* < 0.001, Fig. 4a), nitrite accumulation (*p* < 0.001, Fig. 4b), iNOS expression (*p* < 0.001, Fig. 4c), and IL-6 and MIP-2 (*p* < 0.001, Fig. 5) production compared to those of control cultures. 1,25(OH)_2_D_3_ (100 nM) alone exerted no effects. Cotreating cells with 10 or 100 nM but not 1 nM of 1,25(OH)_2_D_3_ and LPS significantly attenuated levels of ROS production (*p* < 0.001, Fig. 4a). Similarly, cotreatment with 1, 10, or 100 nM of 1,25(OH)_2_D_3_ and LPS for 24 h significantly reduced nitrite accumulation (*p* < 0.001, Fig. 4b) and levels of iNOS expression (*p* < 0.001, Fig. 4c) in a concentration-dependent manner. Moreover, cotreating cells with 100 nM of 1,25(OH)_2_D_3_ significantly reduced LPS-induced IL-6 and MIP-2 production (*p* < 0.001, Fig. 5). Regarding protein expressions, levels of iNOS (Fig. 6a), IL-6, and MIP-2 proteins (Fig. 6b) were significantly reduced in cells exposed to 1,25(OH)_2_D_3_ plus LPS for 24 h. These results indicated that 1,25(OH)_2_D_3_ attenuated LPS-induced iNOS, IL-6, and MIP-2 expressions at both the protein and mRNA levels.

**Fig. 7:**
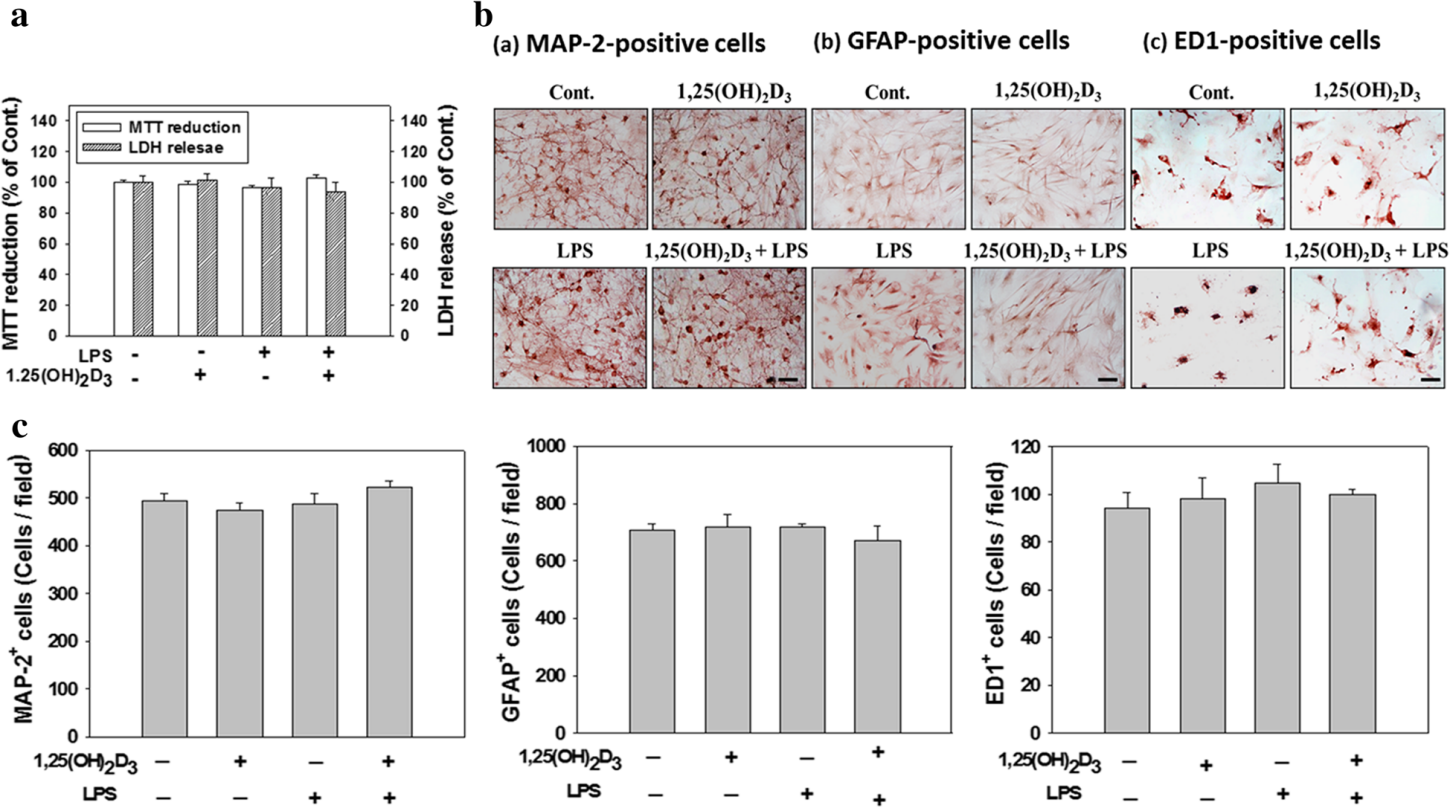
A lack of 1,25(OH)_2_D_3_ affected cell survival and cell death. Cultured cells were untreated (PBS) or treated with 1,25(OH)_2_D_3_ (100 nM), LPS (100 ng/ml), or 1,25(OH)_2_D_3_ plus LPS for 24 h. **a** After treatment, cultured cells were used for an MTT reduction assay. The culture medium was collected for lactate dehydrogenase (LDH) measurement. Cytotoxicity was assayed based on levels of LDH activity in the culture medium. MTT reduction or LDH release data are expressed as a percentage of values relative to control (Cont.) cultures. Data are expressed as the mean ± SEM (*n* = 4 in each group). **b** After treatment, cultured cells were fixed, and then immunocytochemically stained with an antibody against a specific cell marker (MAP-2, GFAP, or ED1). Representative photomicrographs show cultures stained with antibodies to identify neurons (MAP-2-positive cells; *a*), astrocytes (GFAP-positive cells; *b*), and microglia (ED1-positive cells; *c*). *Arrows* indicate activated glial cells. **c** Cell counts of MAP-2-, GFAP-, and ED1-positive cells in cultures exposed to either PBS, 1,25(OH)_2_D_3_, LPS, or 1,25(OH)_2_D_3_ plus LPS for 24 h. Data are the mean ± SEM from three independent experiments

We also monitored cell viability using an MTT reduction assay and confirmed it using LDH measurements. Results from both the MTT reduction assay and LDH measurements showed that the viability of cultured cells exposed to 100 nM 1,25(OH)_2_D_3_ or LPS alone for 24 h did not significantly differ from that of PBS-treated cells (Fig. 7a). The MTT assay revealed that cotreatment with 1,25(OH)_2_D_3_ and LPS non-significantly affected cell viability compared to that of control cultures. Compared to the medium control, no significant differences existed in levels of LDH release (an indicator of cell death) when 1,25(OH)_2_D_3_ and LPS were simultaneously added, indicating that the 1,25(OH)_2_D_3_ concentration used in this study was not toxic to cells. Determination of LDH release and the MTT reduction test might not be sufficient to study cell damage. Therefore, cytotoxicity was further assessed by immunocytochemical staining, and we subsequently counted the number of positive cells with antibodies against MAP-2 (a neuron marker), GFAP (an astrocyte marker), and ED1 (a microglia marker), respectively. Exposure of cortical neuron-glia cultures to either 1,25(OH)_2_D_3_, LPS, or a combination of 1,25(OH)_2_D_3_ and LPS for 24 h did not alter the number of MAP-2-positive cells or the morphology of those cells compared to control cultures. Numbers of GFAP-positive and ED1-positive cells did not significantly differ among control culture and cultures treated with 1,25(OH)_2_D_3_, LPS, or a combination of 1,25(OH)_2_D_3_ and LPS (Fig. 7b, c). Exposure of cells to 1,25(OH)_2_D_3_ alone retained both astrocytes and microglia resting morphologies. However, LPS treatment changed the morphologies of astrocytes and microglia from those representing the resting state to those representing the active state. Activated astrocytes had an enlarged size and a massive accumulation of GFAP-positive filaments; ED1-positive microglia changed from the resting state, which exhibits a small soma and long cytoplasmic processes, to the activated form, which is characterized by an amoeboid appearance. A combination of 1,25(OH)_2_D_3_ and LPS attenuated the LPS-induced morphological changes in astrocytes and microglia. Taken together, our results suggest that the selected concentration of 1,25(OH)_2_D_3_ (100 nM) effectively reduced the LPS-initiated neuroinflammatory response without damaging cells.

### 1,25(OH)_2_D_3_ inhibited LPS-stimulated activation of MAPK signaling

**Fig. 8:**
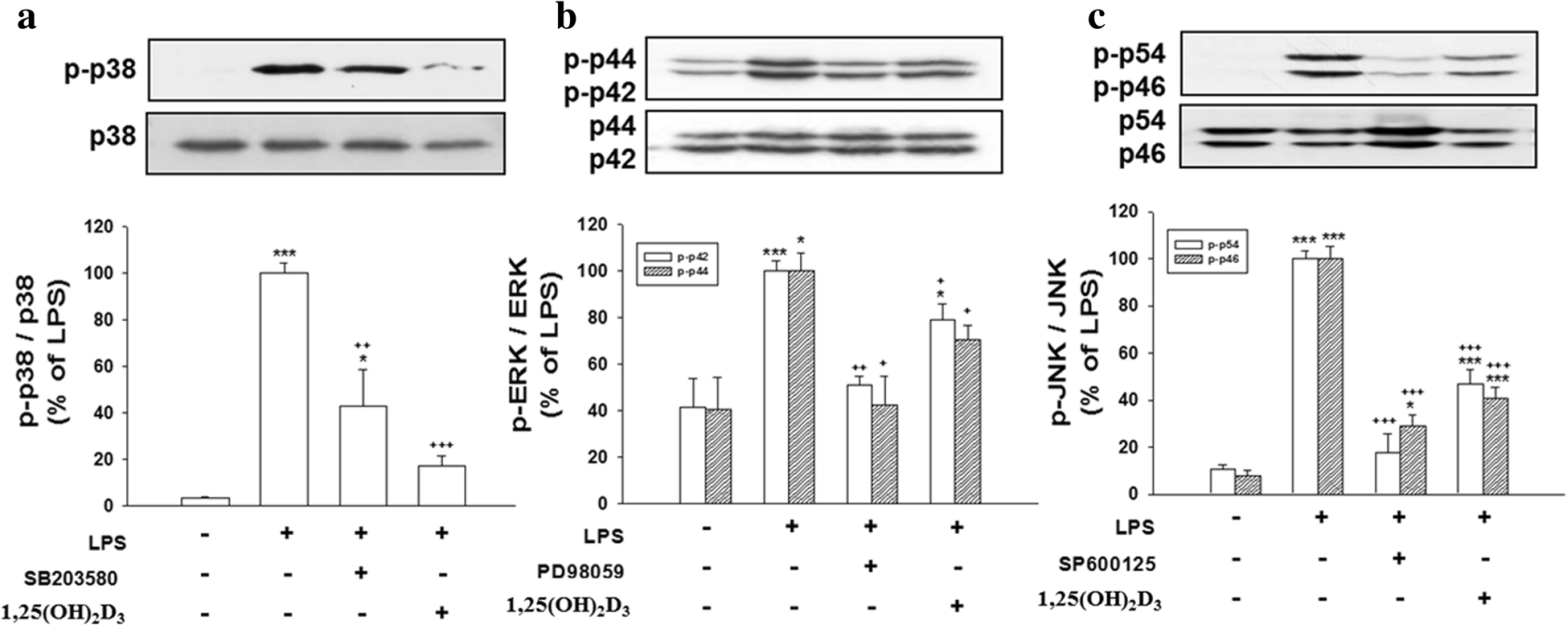
Mitogen-activated protein kinase (MAPK) inhibitors and 1,25(OH)_2_D_3_ inhibited lipopolysaccharide (LPS)-induced phosphorylation of p38, extracellular signal-regulated kinase (ERK), and c-Jun N-terminal kinase (JNK). Cultured cells were treated for 180 min with PBS (control; Cont.) alone, or LPS combined with 20 μM of SB203580, PD98059, SP600125, or 100 nM 1,25(OH)_2_D_3_, and then harvested to conduct Western blot analyses of phosphorylated (p)-p38 (**a**), p-ERK (p42/44) (**b**), and p-JNK (p46/54) (**c**). Levels of p-p38, p-ERK, and p-JNK were respectively normalized to total levels of p38, ERK, and JNK, and then quantified. Data are expressed as the mean ± SEM *(n* = 5 in each group). *p < 0.05, ***p < 0.001 vs. the Cont.; ^+^ *p* < 0.05, ^++^ *p* < 0.01, ^+++^ *p* < 0.001 vs. LPS

To examine whether the antioxidant and anti-inflammatory effects of 1,25(OH)_2_D_3_ were mediated by inhibition of LPS-induced MAPK activation, we examined the effects of simultaneous treatments with LPS for 180 min using various combinations of the p38 inhibitor, SB203580, ERK inhibitor, PD98059, JNK inhibitor, SP600125, and 1,25(OH)_2_D_3_. Western blot results showed that LPS-stimulated phosphorylation of p38, p42/44 (ERK), and p46/54 (JNK) was significantly inhibited by SB203580 (*p* < 0.01, Fig. 8a), PD98059 (*p* < 0.001 for p-p42 and *p* < 0.05 for p-p44; Fig. 8b), and SP600125 (*p* < 0.001 for both p-p46 and p-p54; Fig. 8c), respectively. Cultured cells cotreated with 1,25(OH)_2_D_3_ and LPS exhibited significantly attenuated p-p38 (*p* < 0.001, Fig. 8a), p-p42/44 (*p* < 0.05 for both p-p42 and p-p44; Fig. 8b), and p-p46/54 levels (*p* < 0.001 for both p-p46 and p-p54; Fig. 8c). Activation of the phosphorylation of p38, ERK, and JNK did not significantly differ among control cultures and those treated with MAPK inhibitors or 1,25(OH)_2_D_3_ alone (data not shown). These results suggest that 1,25(OH)_2_D_3_ suppressed the production of LPS-induced inflammatory mediators by inhibiting the p38, ERK, and JNK MAPK pathways.

## Discussion

Glial cells, namely astrocytes and microglia, are the most abundant cells in the brain and play pivotal roles in protecting neurons from harmful insults. Activation of glia following infection or neuronal injury causes neuroinflammation; however, excessive glial activation may exacerbate neuronal damage due to the production of inflammatory molecules. In this study, we used LPS as a stimulant in neuron-glia cultures to initiate a neuroinflammatory response. The excessive production of inflammatory molecules was used as an index of activated glia. In this model, LPS significantly and time-dependently induced the production of inflammatory molecules including NO (reflected by nitrite accumulation), ROS, IL-6, and MIP-2 in cortical neuron-glia cultures. Activation of the p38, ERK, and JNK MAPK pathways is involved in LPS-initiated production of inflammatory molecules. 1,25(OH)_2_D_3_ attenuated LPS-induced nitrite accumulation, iNOS expression, and ROS production in concentration-dependent manners. LPS-induced production of IL-6 and MIP-2 was also significantly reduced by 1,25(OH)_2_D_3_. Furthermore, LPS-elicited phosphorylation of the p38, ERK, and JNK MAPK pathways was decreased by 1,25(OH)_2_D_3_. These results suggest that 1,25(OH)_2_D_3_ attenuates LPS-initiated neuroinflammation through inhibiting MAPK activation in neuron-glia cultures.

We previously demonstrated that astrocytes and microglia are important early sources of proinflammatory mediators in cerebrospinal fluid and brain tissues during bacterial (*Klebsiella pneumoniae*) infection of the central nervous system (CNS) [19]. Brain endothelial cells and pericytes which surround endothelial cells not only contribute to maintenance of the barrier function of the blood–brain barrier but also are immunoactive and can respond to LPS [20–22]. It is possible that LPS may stimulate brain immune cells (e.g., astrocyte and microglia) and other brain cells (e.g., pericytes, endothelial cells, and fibroblasts) resulting in a complex cascade of neuroinflammatory responses [20, 21, 26]. We employed cortical neuron-glia cultures as an *in vitro* model for mimicking the *in vivo* environment in which neurons and glia can interact. The advantages of our *in vitro* model were that (1) cultured cells can bypass any possible physiological feedback interactions *in vivo* to allow direct observation of LPS-induced neuroinflammation in specific cell populations and (2) it provides a simpler system to elucidate cellular mechanisms. However, its limitation is that cell cultures are simply an attempt to provide a simulated microenvironment, and cells are not in their normal physiological or original environment. The cortical neuron-glia cultures in this study consisted of approximately 35 % neurons, 54 % astrocytes, 6 % microglia, 4 % fibroblasts, and a small percentage (<1 %) of other cells including oligodendrocytes, and endothelial cells. The low percentage of other cell types may have been due to the fact that we dissected cerebral cortices and carefully removed blood vessel and meninges during our procedures to prepare the cortical neuron-glia cultures. It is possible that the results of this study can be ascribed to complex interactions between the different cell types found in the cultures. However, considering the relatively poor responses of the small percentage of fibroblasts and other cell types (oligodendrocytes and endothelial cells) to LPS, we propose that glial cells (mainly astrocytes and microglia) were the major responder cells in our culture system.

Glial cells play a role in immune surveillance under normal conditions; however, after a brain injury or exposure to inflammation, glial cells are activated. Under neuroinflammation, glial cells become strongly over-activated through a process that involves phosphorylation of downstream MAPK pathways (e.g., p38, ERK, and JNK) and production of inflammatory molecules [17]. Consistent with our results that treatment of cultured neurons/glia with LPS significantly induced expression of iNOS and the production of NO, ROS, IL-6, and MIP-2, pharmacological inhibition of MAPK pathways by MAPK inhibitors (SB 203580 for p38, PD98059 for ERK, and SP600125 for JNK MAPK) partially attenuated LPS-induced production of inflammatory molecules (ROS, NO, iNOS, IL-6, and MIP-2). A Western blot analysis also confirmed that treatment of cultured neurons-glia with LPS significantly induced phosphorylation of the p38, ERK, and JNK MAPK pathways, indicating that MAPK activation is involved in LPS-induced neuroinflammation. However, sustained overproduction of these inflammatory molecules may further contribute to neuronal damage. Therefore, inhibition of neuroinflammation has become a promising therapeutic target for neuronal injuries.

A dietary vitamin D_3_ (cholecalciferol) deficiency may lead to oxidative stress and elevation of iNOS expression in the brain and further promote cognitive decline in middle-aged and elderly adults [27]. It was reported that inhibition of iNOS expression can cause improvements in clinical signs observed in rats with experimentally produced allergic encephalomyelitis (EAE) treated with 1,25(OH)_2_D_3_ [28]. 1,25(OH)_2_D_3_ also effectively attenuated inflammatory cytokine expressions in the spinal cord and ameliorated EAE in rats [29]. In that model, 1,25(OH)_2_D_3_ treatment significantly reduced Toll-like receptor 8 (TLR8) expression and TLR8 target gene expressions (TNF-α and IL-1β) [29]. Additionally, 1,25(OH)_2_D_3_ provides neuroprotection against 1-methyl-4-1,2,3,6-tetrahydropyridine (MPTP)-induced neuronal injury through inhibition of glial activation and proinflammatory cytokine expression [30]. Those findings suggest that 1,25(OH)_2_D_3_ has important roles in the brain. In this study, we found that LPS-induced ROS production, nitrite accumulation, iNOS expression, and IL-6 and MIP-2 production were significantly reduced after treatment with 1,25(OH)_2_D_3_. It is known that 1,25(OH)_2_D_3_ is one of the important nuclear steroid transcription regulators that can control transcriptions of a large number of genes. 1,25(OH)_2_D_3_ is known to exert its biological functions by directly influencing cellular processes and also by influencing gene expressions through vitamin D response elements (VDREs). We also found that levels of iNOS, IL-6, and MIP-2 mRNA expressions were also significantly attenuated by 1,25(OH)_2_D_3_, indicating that 1,25(OH)_2_D_3_ plays an important role in gene regulation. However, further studies are required to elucidate the role of 1,25(OH)_2_D_3_ in LPS-initiated gene expression.

The apparent lack of cell damage induced by LPS in our cortical neuron-glia culture may have been due to regional differences in the susceptibility to the concentration (100 ng/ml or 0.1 μg/ml) of LPS in a shorter time (24 h) period. Differential neurotoxicity in mixed neuron-glia cultures from various brain regions after treatment with LPS was demonstrated in a previous study [31] in which treatment with LPS at an even higher concentration (1 μg/ml) for a longer time (72 h) did not cause neurotoxicity in cortical or hippocampal neuron-glia culture but caused neurotoxicity in cultures derived from the mesencephalon. In another study using mice cortical neuron-glia culture, neurotoxicity was only observed with a longer exposure time (36 h) when cultures were treated with LPS (1 μg/ml) in combination with interferon (IFN)-γ (5 U/ml) [32]. The concentration of 1,25(OH)_2_D_3_ we used in this study was similar to that in previous reports [33, 34]. In order to exclude the possibility that 1,25(OH)_2_D_3_ attenuated the LPS-induced release of inflammatory molecules because of 1,25(OH)_2_D_3_ toxicity, our results confirmed that 100 nM 1,25(OH)_2_D_3_ was not toxic to cultured neuron-glia cells as revealed by the MTT and LDH assays as well as immunocytochemistry. Because the concentration of 1,25(OH)_2_D_3_ used in our cultures effectively reduced LPS-induced neuroinflammation, we suggest that 1,25(OH)_2_D_3_ supplementation may be a candidate for prevention and treatment of neuroinflammation.

1,25(OH)_2_D_3_ is a neuro-immunomodulator involved in various neurodegenerative and autoimmune diseases [3]. The neuroprotective effects of 1,25(OH)_2_D_3_ have been reported in cultured hippocampal neurons against excitotoxic insults through reduced L-type calcium channel expression [35] and against rotenone-induced neurotoxicity in human neuroblastoma cell line SH-SY5Y cells by enhancing autophagy signaling [36]. Besides the direct neuroprotective effects on neurons, 1,25(OH)_2_D_3_ also reduced neurotoxin-induced dopaminergic neuronal loss by inhibiting of microglia activation and proinflammatory cytokine expression [30]. It is known that 1,25(OH)_2_D_3_ acts via the vitamin D receptor (VDR), a member of the steroid/thyroid hormone superfamily of transcription factors, and the membrane-associated, rapid-response steroid-binding receptor (MARRS), also known as Erp57/Grp58 [3]. The VDR is expressed by glia [4] and neurons [37] in the brain, and not just those participating in the classic actions of vitamin D. The action of 1,25(OH)_2_D_3_ mediates its biological effects by binding to the VDR, which then recruits cofactors to form a transcriptional complex that binds to VDREs in the promoter region of target genes to alter transcriptional cascades within cells [2]. The non-classical action of 1,25(OH)_2_D_3_ is that it binds to the MARRS receptor, located on the cell surface, initiating non-genomic effects [3]. A single-nucleotide polymorphism in the VDR gene can influence the affinity of vitamin D to its receptor and thus may be related to neurodegenerative diseases and neuronal damage by altering vitamin D-mediated pathways [38, 39]. β-amyloid (Aβ) disrupts the vitamin D-VDR pathway and results in iNOS expression in cortical neurons, whereas this effect can be prevented by vitamin D [40]. Moreover, treatment with vitamin D in that model protected neurons by preventing cytotoxicity and apoptosis and also by upregulating the VDR [41]. Those results suggest a potential role for vitamin D-VDR-mediated mechanisms in AD. In this study, we were interested in investigating how 1,25(OH)_2_D_3_ affected LPS-induced neuroinflammatory responses and exploring whether the effects of 1,25(OH)_2_D_3_ were mediated through MAPK signaling pathways. Involvement of MAPK signaling pathways in the production of inflammatory molecules in neurons and glial cells was previously demonstrated [42–44]. The three major MAPK subfamilies of p38, ERK, and JNK are phosphorylated in neurons and glia following LPS treatment [45]. MAPKs are related to LPS signaling in glial cells and lead to iNOS expression and the production of NO and proinflammatory cytokines [45–47]. The current results indicate that the phosphorylation of p38, ERK, and JNK MAPKs occurred after LPS treatment. We demonstrated that 1,25(OH)_2_D_3_ decreased the phosphorylation of p38, ERK, and JNK MAPKs as a result of LPS. This is consistent with a previous report that 1,25(OH)_2_D_3_ reduced macrophage-induced release of chemokines and cytokines by adipocytes and the chemotaxis of monocytes through inhibiting MAPK signaling pathways [48]. Similarly, 1,25(OH)_2_D_3_ diminished LPS-stimulated tumor necrosis factor (TNF)-α and IL-6 release via inhibiting p38 phosphorylation in monocytes/macrophages [49]. Our results suggest that the 1,25(OH)_2_D_3_-mediated suppression of the production of neuroinflammatory molecules may occur through inhibition of MAPK pathways. However, further studies are required to better understand the extents of1,25(OH)_2_D_3_’s anti-inflammatory effects and its involvement in the progression of neuroinflammation.

## Conclusion

Our results suggest that 1,25(OH)_2_D_3_ significantly attenuated the LPS-initiated production of inflammatory molecules by inhibiting p38, ERK, and JNK MAPK signaling in neuron-glia cultures. We suggest that 1,25(OH)_2_D_3_ might be an innovative approach to ameliorating neuroinflammation resulting from various neuronal injuries.

## Abbreviations

1,25(OH)_2_D_3_: 1α,25-dihydroxyvitamin D_3_
ERK: extracellular signal-regulated kinase
GDNF: glial cell line-derived neurotrophic factor
IL: interleukin
iNOS: inducible nitric oxide synthase
JNK: c-Jun N-terminal kinase
LPS: lipopolysaccharide
MAPK: mitogen-activated protein kinase
MARRS: membrane-associated, rapid-response steroid-binding receptor
MIP: macrophage inflammatory protein
NGF: nerve growth factor
NO: nitric oxide
NT3: neurotrophin 3
ROS: reactive oxygen species
VDR: vitamin D receptor
VDRE: vitamin D response element
